# The use of physiotherapy in nursing homes internationally: A systematic review

**DOI:** 10.1371/journal.pone.0219488

**Published:** 2019-07-11

**Authors:** Lindsey Brett, Tim Noblet, Mikaela Jorgensen, Andrew Georgiou

**Affiliations:** 1 Department of Health Professions, Macquarie University, Sydney, NSW, Australia; 2 Australian Institute of Health Innovation, Macquarie University, Sydney, NSW, Australia; 3 Department of Sports and Rehabilitation Sciences, The University of Birmingham, Birmingham, United Kingdom; 4 St George’s University Hospitals NHS Foundation Trust, London, United Kingdom; University of South Australia, AUSTRALIA

## Abstract

**Background:**

Physiotherapy can improve functional ability, prevent falls and reduce pain for older adults in nursing homes. However, there are no legislations or guidelines that specify the parameters of physiotherapy required in nursing homes. With the increasing healthcare demands of ageing populations worldwide, it is important to understand the current use of physiotherapy services to ensure they are both evidence-based and promote equity.

**Objectives:**

(1) When and how are physiotherapy services used by older adults living in nursing homes? (2) What are the factors associated with use of physiotherapy services in nursing homes? (3) How are physiotherapy services in nursing homes documented and monitored?

**Methods:**

Several databases and grey literature (including MEDLINE, PubMed, Pedro and EMBASE) were searched following PRISMA guidelines in March 2018. Searches were limited to English language publications from 1997. Assessment for inclusion, data extraction and quality assessment were completed by two investigators independently using standardised forms. Studies were included if they considered any type of physiotherapy service that involved a qualified physiotherapist (such as exercise, massage and staff education) with older adults (aged 60 years and older) that were primarily permanent residents of a nursing home. Data extracted included proportion of clients that used physiotherapy services, type, frequency and duration of physiotherapy services, and factors associated with physiotherapy service use.

**Results:**

Eleven studies were included. Between 10% and 67% of nursing home clients used physiotherapy services. Factors associated with greater use of physiotherapy services included larger size facilities, and if clients had a physical impairment and mild or no cognitive impairment. Types of physiotherapy services reported were pain management and pressure ulcer management.

**Conclusions:**

Physiotherapy service use in nursing homes varied widely. The development of physiotherapy benchmarks and quality standards are needed to support older adults in nursing homes.

**PROSPERO registration number**: CRD42018082460.

## Introduction

Worldwide, population ageing is increasing pressure on nursing homes to support frail, older adults when they can no longer live at home with family and/or external agency support [1]. Nursing homes are known by various terms worldwide, such as residential aged care facilities, aged care homes, and long term care facilities [2]. For the purpose of this systematic review the term nursing home, defined as “a special purpose facility which provides accommodation and other types of support, including assistance with day to day living, intensive forms of care, and assistance towards independent living, to frail and aged residents” [3]. This term was selected as it was the most common term used in the included studies.

Physiotherapists are commonly utilised in nursing homes to assist with mobility and movement dysfunctions, pain management, falls minimisation, and manual handling education [4]. Existing research demonstrates the benefits of physiotherapy for older adults that live in nursing homes, including improvement in physical performance, functional ability, falls prevention and reduction in pain [5–8]. Healthcare legislation and physical activity guidelines implemented in many countries, including Australia [9], the United Kingdom (UK) [10], and the United States of America (USA) [11], recommend the use of physiotherapy and exercise to help restore and maintain the function of older adults living in nursing homes [12]. However, they do not specify the minimum physiotherapist staffing levels, type of physiotherapy, or frequency and duration of physiotherapy required to achieve this recommendation.

It is important to understand how physiotherapy is currently used and monitored to ensure services are evidence-based and equitably available in nursing homes. This systematic review aimed to assess and synthesise the current international literature evaluating the use of physiotherapy in nursing homes by considering:

1. When and how physiotherapy services are used by older adults living in nursing homes (e.g. type, duration, frequency and funding)?
2. What are the factors associated with use of physiotherapy services?
3. How are physiotherapy services in nursing homes documented and monitored (e.g. outcome indicators and effectiveness)?

## Methods

To ensure transparency and reproducibility, and reduce reporting bias this systematic review protocol was registered with PROSPERO (registration number: CRD42018082460, S1 File) [13], and was reported in accordance with the PRISMA statement (S1 Table) [14, 15].

### Identification and selection of studies

To build the search strategies, relevant keywords were identified through an initial search on MEDLINE. Detailed searches were conducted across several databases (MEDLINE via Ovid, PubMed, CINAHL, Cochrane Library, AMED, Pedro and OTseeker) and grey literature sources in March 2018 using pre-determined search criteria (S1 Fig). Using a systematic grey literature search template developed in a Canadian study [16], search strategies were conducted on grey literature databases (e.g. EMBASE, ProQuest Dissertations and Thesis Global), Google search engine and targeted websites (e.g. Australian Physiotherapy Association, AGILE–Chartered Physiotherapists working with Older People, International Federation on Ageing, and National Aged Care Alliance) to reduce the risk of publication bias [15]. All searches were limited to the English language as there was no funding available for translation. Publications from 1997 were searched as major legislation and frameworks related to aged care reform were implemented in many countries from 1997 [9, 10, 17, 18]. Pearling of the including studies was completed to help ensure all appropriate studies were identified.

**Fig 1:**
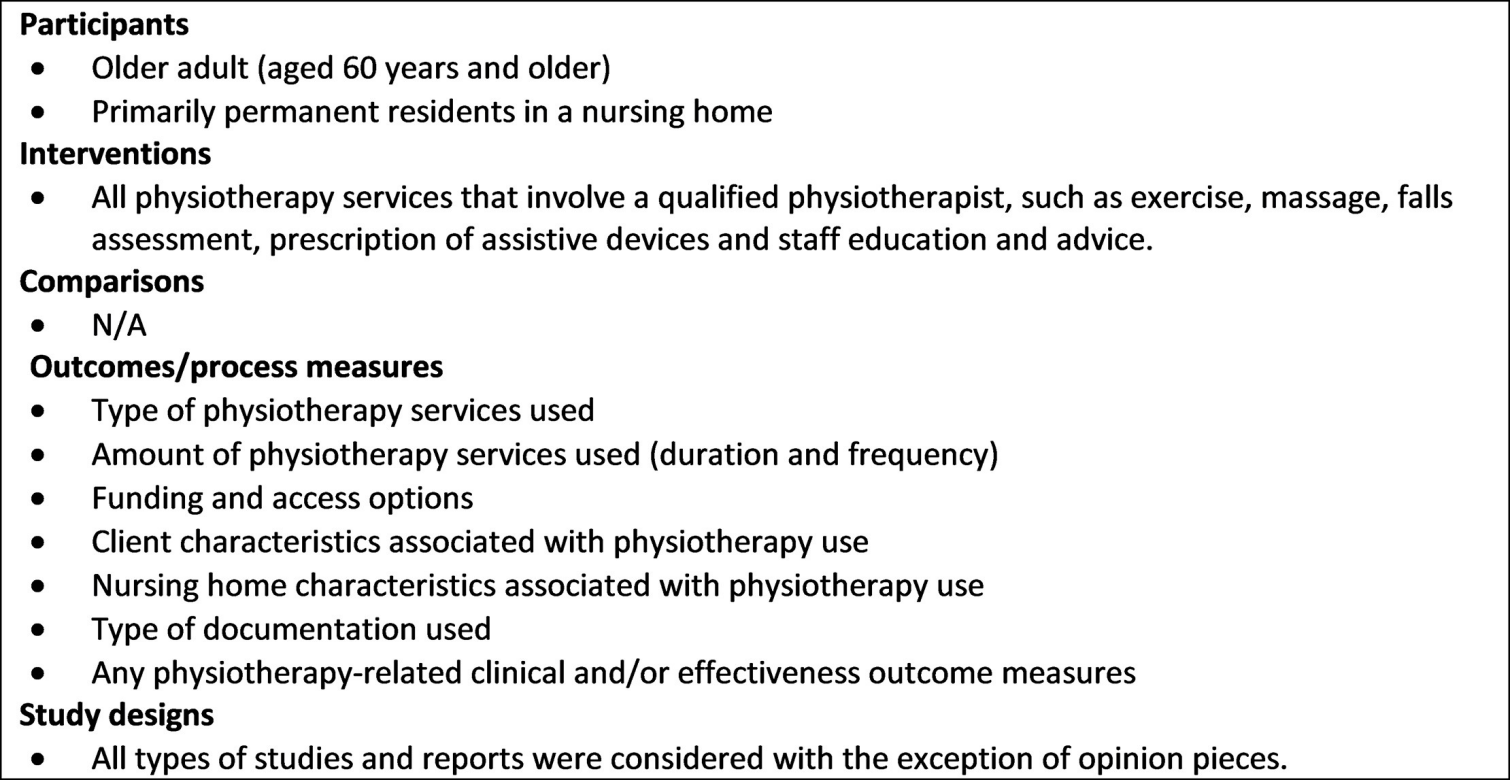
Inclusion criteria (PICOS).

Assessment for inclusion was completed independently by two investigators who were blinded to journal and author names. Studies were included if they self-reported physiotherapy services that involved a physiotherapist/physical therapist (commonly used terms internationally) with older adults that were primarily permanent residents of a nursing home. This could include physiotherapy services that also involved physiotherapy assistants, as long as there was also a physiotherapist involved in the service proivsion. The PICOS framework was used to develop the inclusion criteria (Fig 1) [14]. The initial stage involved review of the title and abstract against the inclusion criteria. The full manuscript of the papers selected were then assessed against the inclusion criteria. Following an independent selection process, the two investigators conferred and resolved discrepancies between their final selections of papers with the help of a third investigator. To reduce evidence selection bias, the references of all papers that met the inclusion criteria were checked for potential papers that may have been missed during database searches [15].

### Assessment of study characteristics and risk of bias

Quality and risk of bias were assessed independently by two investigators using the Joanna Briggs Institute (JBI) Appraisal Checklist for Analytical Cross-Sectional Studies (S2 Fig) [15, 19]. This appraisal tool consists of eight questions and allows for the removal of irrelevant questions as required. One question (question seven) was removed for this systematic review as it focused on the validity and reliability of outcome measures, which had not been reported in any of the included studies. In all remaining questions, if the answer was unclear based on the detail provided the question was marked as ‘no’, if the question was not applicable to a study it was documented as ‘N/A’ and the total score adjusted. The two investigators discussed their quality assessments, and a third investigator was available to resolve any debates.

### Data extraction and analysis

Two investigators used a standardised data extraction form developed for this systematic review based on two JBI data extraction tools (S3 Fig) [19]. The investigators pre-tested and became familiar with the form prior to data extraction to ensure consistency during the process, which were cross-checked on completion. Descriptive analysis was used to quantify and compare the study characteristics: type, frequency and duration of physiotherapy services used in nursing homes, and client and nursing homes characteristics associated with physiotherapy use.

### Summary measures and synthesis of results

The study characteristics and main findings of the included studies were tabulated. Studies were grouped by sample (nursing home clients, nursing homes and physiotherapists that worked in nursing homes) to allow for comparison between studies. Factors associated with the use of physiotherapy were categorised according to the Andersen Healthcare Utilisation Model [20]. This model suggests healthcare utilisation is based on a number of complex, interrelated social behaviour factors: (i) the predisposition of an individual to use services (predisposing factors), (ii) their ability to secure services (enabling factors), and (iii) their need for such services (need factors) [20].

## Results

### Flow of studies

**Fig 2:**
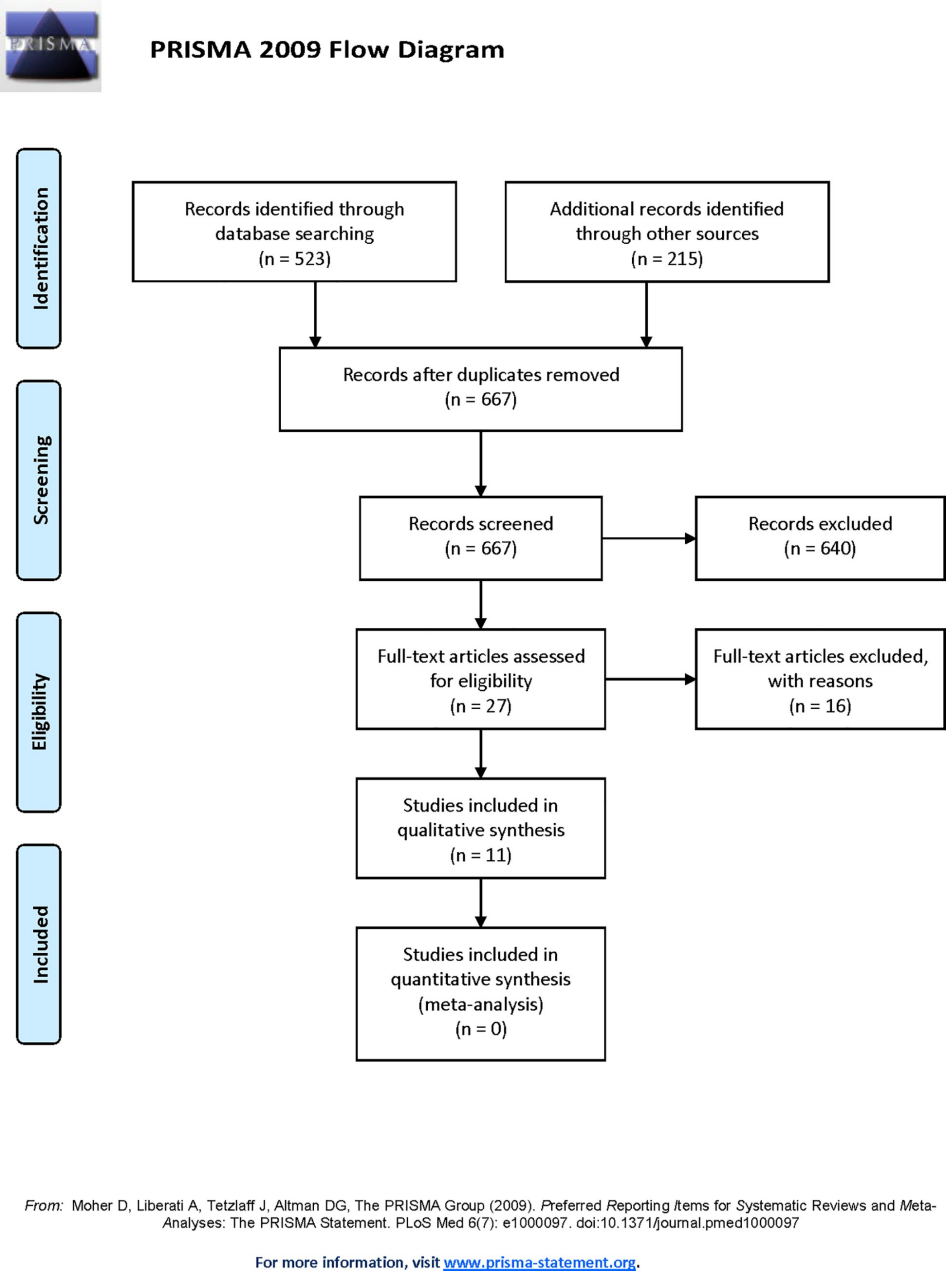
PRISMA flow diagram of studies through the review [14].

There were 669 papers identified through the database searches. Following removal of duplicates, 460 papers were reviewed for inclusion by title and abstract. Twenty-seven potential papers required full paper review. Following completion of the review process 11 papers were included in this systematic review (Fig 2) [21–31].

### Quality of studies and risk of bias

The majority of the included studies (n = 8) met at least 60% of the criteria outlined by the JBI quality appraisal checklist (Table 1) [22–27, 29, 31]. Two of the studies provided sufficient detail to address all of the checklist criteria [25, 26]. The majority of the studies used objective, standard criteria for measurement (82%), appropriate statistical analysis (82%), and when applicable identified confounding factors (100%). Some studies were at risk of bias due to the lack of detail provided to sufficiently describe the inclusion criteria (45%), validity and reliability of the measurement used (55%), and when applicable the strategies adopted to deal with confounding factors (47%).

**Table 1:** JBI critical appraisal scores of included studies.

Study	Methodological item^#^							Total
	1	2	3	4	5	6	7	
APA [30]	N	N	N	Y	N/A	N/A	N	1*
Barodawala, Kesavan and Young [21]	N	N	N	Y	N/A	N/A	Y	2*
Berg et al [22]	N	Y	Y	N	Y	Y	Y	5
Bhuyan et al [27]	Y	Y	N	Y	Y	Y	Y	6
Buchanan et al [23]	Y	Y	Y	Y	Y	N	Y	6
De Boer et al [28]	N	Y	N	N	Y	Y	Y	4
Harrison and Lemke [31]	Y	Y	N	Y	N/A	N/A	Y	4*
Kinley et al [24]	N	N	Y	Y	N/A	N/A	Y	3*
Leemrijse et al [25]	Y	Y	Y	Y	Y	Y	Y	7
Mc Arthur et al [26]	Y	Y	Y	Y	Y	Y	Y	7
O’Dea, Kerrison and Pollock [29]	Y	Y	N	Y	N/A	N/A	N	3*

*Maximum total score possible was five; items 5 and 6 related to confounding factors and not applicable to study.

^#^Methodological items

1. Inclusion criteria clearly defined

2. Study subjects and setting detailed

3. Exposure measured in a valid and reliable way

4. Objective, standard criteria used for measurement

5. Confounding factors identified

6. Strategies to deal with confounding factors stated

7. Appropriate statistical analysis used

### Characteristics of studies

Table 2 summarises the characteristics and main findings of the included studies. Studies were from the USA (n = 4) [22, 23, 27, 31], UK (n = 3) [21, 24, 29], Netherlands (n = 2) [25, 28], Australia (n = 1) [30], and Canada (n = 1) [26]. One of the USA studies involved collection of data from USA, Italy, Japan, Iceland and Denmark [22]. Study designs included surveys (n = 6) [21, 27–31], examination of client notes and assessments (n = 4) [22–24, 26], and interviews with clinicians (n = 1) [25]. The three types of sample populations identified in the included studies were: (i) clients that lived in nursing homes (n = 6), (ii) nursing homes (n = 3), and (iii) physiotherapists that worked in nursing homes (n = 2). Three studies focused on a specific population/time within the sample groups: nursing home clients’ final six months of life [24], male Veterans Health Administration clients [23], and physiotherapists that provided pressure ulcer management [31]. In all studies the majority of the clients living in nursing homes were female, except the study which focused on male veterans. The mean age of clients ranged between 71–85 years (reported in three studies) [23–25], in two studies the majority of clients were classified as 85 years or older [22, 26].

**Table 2:** Summary of included studies.

Study	Country	Design	Participants	Main findings
**Physiotherapy use by nursing home clients**				
Barodawala, Kesavan and Young [21] (2001)	UK (excluding Northern Ireland)	Cross-sectional postal and telephone survey with Matron/nurse-in-charge Data collection period: 6 months	n = 12 588 (346 nursing homes) Response rate: 97% Median number (interquartile range) of clients per nursing home: • Postal survey: 35 (25–47) • Telephone survey: 31 (20–44)	*Physiotherapy use:* • At the time of the survey: 10.4% clients (postal and telephone) • Within the previous 6 months: 11.6% clients (postal) *Factors associated with physiotherapy:* • Employed private physiotherapist *Funding and access to physiotherapy:* • 75% relied on GP referral for physiotherapy (postal) • 25% had a regular physiotherapist that attended 1–4 sessions per week (postal) • nursing homes with regular private physiotherapists: 17.3% (postal and telephone) • nursing homes with regular NHS funded physiotherapists: 7% (postal)
Berg et al [22] (1997)	USA, Denmark, Iceland, Italy and Japan	Cross-sectional examination of MDS Ax completed by experienced RNs Data collection period: 7 days	n = 280 540 (USA: 273 491, Denmark: 3 451, Iceland: 1 254, Italy: 1 089, Japan: 1 255) Client characteristics: • Female: 73% • Aged 85 years or older: 46% • LoS since admission greater than 90 days: 76% (data not available for Italy)	*Physiotherapy use*: • All countries: 14% • By country: Iceland 32%, Japan 20%, Denmark 19%, USA 14%, Italy 12% *Factors associated with physiotherapy*: • Poor ADL score and good CPS score
Buchanan et al [23] (2004)	USA	Exploratory examination of MDS Ax completed by trained clinicians Data collection period: 7 days	n = 166 933 (7 730 male VHA clients, 159 203 all other male clients) *Specific client group*: male VHA in nursing homes *Client characteristics (mean):* • Age: VHA: 72.1, all other: 71.6 • White (not Hispanic) ethnicity VHA: 78.6%, all other: 77.2%	*Physiotherapy use:* • Min of physiotherapy per week ○ Male VHA clients: mean = 39.6 ±81.3, median = 0, 75^th^ percentile = 30 ○ All other male clients: mean = 80.4 ±134.4, median = 0, 75^th^ percentile = 150 ○ (mean: p <0.01) *Factors associated with physiotherapy:* • Non-VHA status
Kinley et al [24] (2014)	UK	Cross-sectional examination of nursing home notes by researchers (part of a large cluster RCT) Data collection period: 6 months	n = 2 444 (38 nursing homes) *Specific time point*: last six months of life for clients living in nursing homes *Client characteristics (mean):* • Female: 61% • Age: 85 years • LoS since admission: 20 months • Medical diagnoses: 4 • Diagnosis of dementia or cognitive impairment: 79%	*Physiotherapy use:* • Total sample: 12% • Per nursing home: range 0–56, mean 1
Leemrijse et al [25] (2007)	Netherlands	Cross-sectional interviews (questions developed from RAI Ax) with nursing homes physicians and physiotherapists Data collection period: 6 months	n = 600 (15 nursing homes) *Client characteristics (mean):* • Female: 65% (n = 391) • Age: 81.53 (SD 8.24) • LoS since admission: 3.13 years (SD 3.56) • Most common medical diagnoses Dementia: 43.3%, stroke: 24.2% • Medical diagnoses: 3.52 (SD 2.26) • Limited mobility and self-care ability: 44.3% *nursing home characteristics:* • Type Somatic only: 2, combined somatic and psychogeriatric: 13 • Location Urban: 9, rural: 6	*Physiotherapy use:* • Total sample: 67.3% • Per nursing home: 69%, range 39–93%, interquartile range 34.2 • Min of physiotherapy per week: mean (SD) = 55(41), range = 34–88, interquartile range = 15 *Factors associated with physiotherapy use:* • Male gender, greater number of co-morbidities, shorter LoS since admission (years), residing in a somatic ward, more FTE physiotherapists present, admitted for rehabilitation *Factors associated with greater amount of physiotherapy:* • Admitted for rehabilitation, post THR, nursing homes that provided both somatic and psychogeriatric care, main limitation/impairment associated with mobility and self-care, fewer impairments and limitation in activities
Mc Arthur et al [26] (2015)	Canada	Cross-sectional, population-based, examination of RAI Ax by researchers Data collection period: 7 days	n = 87 869 *Client (that received physiotherapy) characteristics:* • Female: 61.1% (n = 6 115) • Aged 85 years and older: 55.7% (n = 5 570) • LoS since admission <365 days: 8.5% (n = 847), 365–730 days: 16% (n = 1 604), >730 days: 75.5% (n = 7 554) • Most common medical diagnoses Arthritis: 32.1%, stroke: 20.3%, osteoporosis: 18.2% • Moderate to severe ADL impairment: 77.8% (n = 7 783) • Moderate to severe cognitive impairment: 57.6% (n = 5 764)	*Physiotherapy use:* • Any amount of physiotherapy: 11.4%, range = 5.8%-29.5% • 64–88% did not receive any physiotherapy • Min of physiotherapy per week ○ 45 or more, on three days or more: 1.9%-7.1% ○ 150 or more, on five days or more: 0.04%-0.7% *Factors associated with physiotherapy use:* • Female gender, younger age, no cognitive impairment (CPS = 0), no depression (DRS = 0), LoS since admission <365 days, potential for improvement (self and staff rated), certain provinces or territories, improved clinical status from last Ax, medium-high falls risk trigger in care system, urinary incontinence trigger in care system, ADL impairment (ADL Hierarchy score >0), pain (pain score >0), diagnosis of MS, Parkinson’s, stroke, pneumonia, any fracture, hip fracture or osteoporosis, experiencing an acute event
**Physiotherapy use by nursing homes**				
Bhuyan et al [27] (2017)	USA	Cross-sectional examination of data collected from the 2010 NSRCF conducted by the Centers for Disease Control and Prevention Data collection period: 12 months	n = 2 302; weighted sample, 31 134 Client characteristics (mean): • Female residents: 68.2% • White residents: 88.5% *nursing home characteristics (weighted):* • Number of beds (size) 4–10 (small): 49.5%, 11–25 (medium): 15.9%, 26–100 (large): 27.8, >100 (extra-large): 6.7% • Ownership Private for-profit: 82.4%, private non-profit, state, county or local government: 17.6%	*Physiotherapy use:* • Total sample: 43.9% (weighted) *Factors associated with physiotherapy use:* • nursing home with high percentage of white residents, higher PCA HPPD, licenced director, nursing home that used volunteers, large (26–100 beds) or extra-large (≥100 beds) nursing homes, Medicaid certified, private for-profit ownership
De Boer et al [28] (2007)	Netherlands	Telephone survey with allied healthcare managers Data collection period: not stated	n = 88 Response rate 88% *nursing home characteristics*: • Type of care Somatic: 15% (n = 13), combined somatic and psychogeriatric: 85% (n = 75) • Number of beds/places 0–100: 14.8%, 101–200: 38.6%, >200: 46.6%	*Physiotherapy use:* • Total sample: 99% • Average physiotherapist availability rate: 2.16 FTE per 100 beds/places *Factors associated with physiotherapy use:* • No associations found with investigated factors (size of the nursing home, location of nursing home, presence of specialised wards/units, number of beds/places within specialised wards/units)
O’Dea, Kerrison and Pollock [29] (2000)	UK	Telephone survey with nursing home managers Data collection period: 12 months	n = 49 (1 808 beds, 1 541 clients) Response rate 96% *nursing home characteristics*: • Type of care Frail elderly: 39% (n = 19), elderly mentally infirm: 20% (n = 10), elderly mentally infirm and frail elderly: 8% (n = 4), elderly mentally infirm and frail elderly as well as other client groups: 24% (n = 12) • Mean beds: 37 • Ownership Private: 88% (n = 43), voluntary: 12% (n = 6)	*Physiotherapy use:* • Total sample: 76% *Factors associated with private physiotherapy use:* • Larger nursing homes (mean beds = 41) *Funding and access to physiotherapy:* • nursing homes with regular physiotherapy: 20.4% (n = 10) ○ FT physiotherapists: 2 ○ Physiotherapists that attended one to three sessions per week: 8 • NHS only 41% (n = 20), private only 22% (n = 11), NHS and private 12% (n = 6), no service 24% (n = 12)
**Physiotherapy use by nursing home physiotherapists**				
APA [30] (2014)	Australia	Online survey completed by nursing home physiotherapist that were members of the APA or PBA Data collection period: not stated	n = 370 Question response rates: 4%-62% *Physiotherapist characteristics*: • Location NSW: 49%, Victoria: 18%, Queensland: 12%, WA: 8%, SA: 6%, Tasmania: 4%, ACT: 3%, NT: 0% • Employment Permanent: 32%, contract: 63%, other: 5% *nursing home characteristics:* • Type of care High level care: 65%, low level care: 35% • Mean beds: 86	*Physiotherapy use:* • Physiotherapist time spent ○ Clinical work: 66.33% ○ Pain management treatments: 46% ○ Non-pain management treatments: 25% ○ Paperwork: 27% *Funding and access to physiotherapy:* • Physiotherapists work an average of 19 hours per week, per nursing homePhysiotherapist opinions on use and funding also reported
Harrison and Lemke [31] (2004)	USA	Postal survey completed by nursing home physiotherapists Data collection period: not stated	n = 68 Response rate 68.7% *Specific service type*: pressure ulcer management *Physiotherapist characteristics*: • Role Director of rehabilitation/ physiotherapy: 35.3% (n = 24), physiotherapy supervisor: 26.5% (n = 18), staff physiotherapist: 19.1% (n = 13), other: 1.5% (1) • Location: Arizona, USA • Employment status Employee: 63.2% (n = 43), contractor through agency: 14.7% (n = 10), independent contractor: 2.9% (n = 2)	*Physiotherapy use:* • Physiotherapists currently treating clients for pressure ulcer management: 70.6% • Average number of clients with pressure ulcers treated by physiotherapists per week ○ 1–5: 64.7% (n = 44) ○ 6–10: 4.4% (n = 3) ○ 10–15: 1.5% (n = 1) Most used and available modalities also reported

RAI: Resident Assessment Instrument, Ax: Assessment, ADL: Activities of Daily Living, LoS: Length of Stay; MS: Multiple Sclerosis, UK: United Kingdom, RCT: Randomised Controlled Trial, THR: Total Hip Replacement, MDS: Minimum Data Set, USA: United States of America, VHA: Veterans Health Administration, NHS: National Health Service, GP: General Practitioner, RN: Registered Nurse, CPS: Cognitive Performance Scale, DRS: Depression Rating Scale, NSRCF: National Survey of Residential care Facilities, PCA HPPD: Personal Care Aide Hours Per Patient per Day, SD: Standard Deviation, FTE: Full Time Equivalent, FT: Full Time, APA: Australian Physiotherapy Association, PBA: Physiotherapy Business Australia, NSW: New South Wales, WA: Western Australia, SA: South Australia, ACT: Australian Capital Territory, NT: Northern Territory.

The data collection period was reported in eight of the 11 studies: seven days (n = 3), six months (n = 3) or 12 months (n = 2). The studies that collected data over a seven day period used either the Minimum Data Set (MDS) or Resident Assessment Instrument (RAI) 2.0, which are seven-day assessments completed within 14 days of admission of an older adult to a nursing home in the USA or Canada respectively [22, 23, 26]. The studies that used longer data collection periods were based on information from self- reported surveys or review of client notes. Response rates were reported in five of the six survey studies and varied between 69% for physiotherapists in the USA to 97% for matrons/nurse-in-charge in the UK [21, 31].

### Summary measures and synthesis of results

Process measures used (type, duration, frequency, funding, and factors associated with physiotherapy use) were based on self-reported data collected from surveys and interviews (n = 7), or reviews of clinical assessments and notes (n = 4).

#### Use of physiotherapy services

Seven studies reported either the number of clients or nursing homes that used physiotherapy services [21, 22, 24, 26–29]. The mean proportion of clients that used physiotherapy services in nursing homes varied across countries, from 10% in the UK to 67% in the Netherlands [21, 25]. The majority of the studies that examined physiotherapy use by clients (n = 4) found less than 25% of nursing home clients used physiotherapy services [21, 22, 24, 26]. When examined by nursing home (n = 3), the percentage of nursing homes that used physiotherapy services was 44% in the USA [27], 76% in the UK [29], and 99% in the Netherlands [28].

#### Duration and frequency of physiotherapy in nursing homes

Three studies reported the number of minutes per week clients used physiotherapy services [23, 25, 26]. Studies from the USA and Netherlands found the mean time per week ranged from 40 (±81) to 80 (±134) minutes [23, 25]. A study from Canada used time categories from the Resource Utilisation Groups version III [32], to quantify the frequency and duration of physiotherapy service use; 45 minutes over three days or 150 minutes over five days [26]. This study found 2–7% of clients used physiotherapy services for more than 45 minutes over three days per week, and less than 1% used more than 150 minutes of physiotherapy services over five days per week.

#### Type of physiotherapy in nursing homes

Two studies considered type of services; both collected data from physiotherapists working in nursing homes [30, 31]. Australian physiotherapists’ time was spent completing pain management services (46%), non-pain management services (25%) or paperwork and administrative tasks (27%) [30]. Seventy-one percent of physiotherapists in Arizona, USA completed pressure ulcer management as part of their regular duties [31]. The most common treatment modalities were whirlpool, ultrasound, electrical stimulation, high volt pulsed current, and vacuum closure system [31].

#### Funding of physiotherapy in nursing homes

Data related to funding of physiotherapy services in nursing homes were collected in two UK studies [21, 29]. One study used a stratified sample from across the UK (excluding Northern Ireland), and found regular physiotherapy services were more often privately funded (18%) compared to publicly funded through the National Health Service (NHS) (7%). They also found 75% provided ad hoc physiotherapy services via General Practitioner (GP) referral [21]. In contrast, 41% of the nursing homes reviewed in the study conducted in the south-eastern region of England used only NHS physiotherapists, 22% used only private physiotherapists, 12% used both, and 24% had no physiotherapy services [29].

#### Client and nursing home factors associated with physiotherapy use in nursing homes

Seven studies reported an array of factors (n = 37) that could potentially affect the use of physiotherapy services in nursing homes (Table 3) [21–23, 25–28]. There was very little overlap between studies; only four factors (gender, size of nursing home, length of stay since admission, location) were considered in more than one study [20, 33]. The Anderson Healthcare Utilisation Model was used to categorise the factors associated with use of physiotherapy services in nursing homes (Table 3) [20, 33]. The predisposing factor group was the smallest (n = 8) and consisted of predominately ‘fixed’ factors (e.g. gender, age, race) [23, 25–27]. Enabling factors were commonly associated with workforce and nursing home characteristics [21, 25–27]. Need factors identified related to clients’ health status [22, 25, 26]. One study considered factors related to greater use of physiotherapy services, and found a positive association with admission for rehabilitation, post total hip replacement, nursing homes that provide combined care (somatic and psychogeriatric care), client’s main limitation being mobility or self-care related, and clients with fewer impairments and limitations in activities [25].

**Table 3:** The Anderson Healthcare Utilisation Model: Factors associated with physiotherapy use in nursing homes.

Predisposing factors	Enabling factors	Need factors
• Male [25]^◆^ • Female [26]*^⇞^ • Younger age [26]*^⇞^ • Non-VHA status [23]^⇞^ • Self-rated potential for improvement [26]^⇞^ • Staff-rated potential for improvement [26]^⇞^ • High percentage of white clients [27]^⇞^ • Geographical location [26]^⇞^	• Shorter LoS since admission (years) [25]*^◆^ • LoS since admission <365 days [26]*^⇞^ • Residing in somatic nursing home/unit [25]^◆^ • Improved clinical status from last Ax [26]^⇞^ • More FTE physiotherapists [25]^◆^ • Employed private physiotherapist [21]^#^ • Greater PCA HPPD [27]^◆^ • Licenced director [27]^◆^ • Nursing home that used volunteers [27]^⇞^ • Large (26–100 beds) nursing home [27][Table-fn t003fn005] • Extra-large (≥100 beds) nursing home [27][Table-fn t003fn005] • Private for-profit ownership [27]^⇞^ • Medicaid certified nursing home [27]^⇞^ • Medium-high falls risk trigger in care system [26]^⇞^ • Urinary incontinence trigger in care system [26]^⇞^	• ADL impairment (ADL Hierarchy score >0) [26]^⇞^ • Poor ADL score and good CPS score [22]^#^ • Pain (pain score >0) [26]^⇞^ • Greater number of co-morbidities [25]^◆^ • No cognitive impairment (CPS = 0) [26]*^⇞^ • No Depression (DRS = 0) [26]*^⇞^ • Diagnosis of MS [26]^⇞^ • Diagnosis of PD [26]^⇞^ • Diagnosis of stroke [26]^⇞^ • Diagnosis of pneumonia [26]^⇞^ • Diagnosis of any fracture [26]^⇞^ • Diagnosis of hip fracture [26]^⇞^ • Diagnosis of OP [26]^⇞^ • Experiencing an acute event [26]^⇞^ • Admitted for rehabilitation [25]^◆^

CPS: Cognitive Performance Scale, DRS: Depression Rating Scale, VHA: Veterans Health Administration, LoS: Length of Stay, PCA HPPD: Personal Care Aide Hours Per Patient per Day, FTE: Full-time Equivalent, ADL: Activities of Daily Living, MS: Multiple Sclerosis, PD: Parkinson’s Disease, OP: Osteoporosis

*positive complimentary factor presented in the original study

^◆^p<0.05

^⇞^p<0.01

^
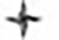
^p<0.001

^#^p value not provided

#### Documentation and monitoring of physiotherapy services in nursing homes

None of the included studies reported physiotherapy outcomes, or the means of documentation and monitoring of physiotherapy services in nursing homes.

### Additional analyses

Due to heterogeneous characteristics of the data collected in the included studies (e.g. different study designs, sample population, process measures etc) a meta-analysis was not possible.

## Discussion

This systematic review identified 11 studies which investigated the use of physiotherapy services in nursing homes in the UK, USA, Denmark, Iceland, Italy, Japan, Netherlands, Canada and Australia. The use of physiotherapy was quantified at either client level [21–26], physiotherapist level [30, 31], or facility level [27–29]. A wide range of variables (e.g. proportion of clients that used physiotherapy services, physiotherapist hours per week, type of pressure ulcer management modalities) were reported across the studies which limited comparability of the included studies. A number of gaps in what is known about physiotherapists’ adherence to evidence-based practice in nursing homes were identified: specific details of type, frequency, duration and funding of physiotherapy services in nursing homes, the physiotherapy outcome measures used in nursing homes, and processes for documenting and monitoring effectiveness of physiotherapy services.

### International utilisation of physiotherapy services

Nursing homes in the Netherlands demonstrated the greatest use of physiotherapy services; 67% of clients accessed physiotherapy in one study [25], and 99% of nursing homes in another [28]. Unlike many other countries, nursing homes in the Netherlands aim to discharge clients, of which 44% return home [28]. The greater use of physiotherapy services could be attributed to the strong rehabilitation approach adopted in this country’s nursing homes where allied health input is considered essential [28]. Funding is not a barrier in the Netherlands as all expenses for nursing home clients are covered regardless of personal financial resources [34].

In the USA, UK, Canada, Denmark, Iceland, Italy and Japan, use of physiotherapy services was low, on average 12% of clients [21, 22, 24, 26], and 60% of nursing homes used physiotherapy services [27, 29]. Some nursing homes in Canada, UK, Denmark, Italy and Japan did not use physiotherapy services at all [22, 24, 26, 29]. While nursing homes in the Netherlands employ specially trained physicians (one full time doctor per 100 beds), the number of GPs and level of attendance varies greatly in other countries, which can lead to reduced referrals to external services like physiotherapy which can require GP initiation [35]. The low utilisation of physiotherapy services in some countries suggests that many clients could be missing out on beneficial care, resulting in poorer health outcomes. The development of quality indicators that require nursing homes to report rates of physiotherapy use, and legislation that codifies evidence-based recommendations around physiotherapy service use may help to ensure nursing homes provide appropriate care to those clients that require it.

European guidelines recommend a personalised, multimodal exercise program at least twice a week for 35 to 45 min per session for every older adult living in a nursing home that has no contraindications to exercise [12]. It is unclear from this systematic review if current physiotherapy services adhere to these (and similar) recommendations, or if physiotherapy services used in nursing homes are clinically effective. Only two studies provided insight into physiotherapy service type. An Australian study provided very broad categories (e.g., pain management, non-pain management and paperwork) [30], whilst an American study reported specific modalities of pressure ulcer management [31]. The focus on pain management services in the Australian study is likely due to the current aged care funding model which only funds physiotherapists to provide massage and electrotherapy for pain management [30], despite the lack of endorsement of these strategies by current evidence-based recommendations for chronic pain [36]. The American study focused on pressure ulcer management [31], which is a service predominately practiced by physiotherapists in the USA only [37]. Among the few studies that considered duration of physiotherapy services in nursing homes (n = 3), weekly duration was lower than that recommended by research and exercise guidelines [23, 25, 26]. The observed differences in physiotherapy services across the included countries are likely influenced by social policies and context specific to each country, for example different models of healthcare funding, nursing home standards and physiotherapist skillset.

Modifiable enabling factors (Anderson Healthcare Utilisation Model) related to workforce, such as employment of physiotherapists that provide regular services [21, 25], and greater numbers of personal care aides [27], were positively associated with physiotherapy service use and suggest the need for government regulation of minimum staffing levels in nursing homes to ensure optimum care. Many older adults that live in nursing homes have some form of cognitive and/or mood impairment [38]. This systematic review found need factors such as a cognitive or mood impairments were negatively associated with physiotherapy service use, as were older age and a longer length of stay, which suggests current practice potentially prevented some clients from accessing physiotherapy services [22, 26]. Identification of strategies to overcome barriers to physiotherapy use amongst specific populations within nursing homes is needed, particularly as there is strong evidence that physiotherapy services can improve mood and slow the progression of cognitive decline [8].

With aged care systems and workforce needs under review worldwide, it is imperative to ensure robust, evidence-based benchmarks and standards are used to monitor, enhance and improve the quality and scope of physiotherapy services for older people in nursing homes. This could include consideration of emerging advanced physiotherapy skills, such as physiotherapist prescribing in the UK, which may enhance practice in the aged care field, although appropriate support and planning is needed if utilisation is to be successful [39, 40]. Allowing physiotherapists to develop their skillset through the provision of advanced services in nursing homes could encourage more physiotherapists to work and remain in aged care, which is vital in ensuring sufficient workforce levels to care for the growing population of older adults.

One of the aims of this systematic review was to consider how physiotherapy services were documented and monitored in nursing homes; none of the included studies provided data on this topic. The development of efficient documentation processes (e.g. information technology programs) and use of evidence-based benchmarks to monitor physiotherapy services could benefit all nursing home stakeholders. If clients and their families had a better understanding of what physiotherapy services were available they could make informed-decisions about their care. Physiotherapists could be guided by evidence-based benchmarks to develop their own practice. Nursing home managers could accurately monitor workforce levels and service provisions through review of clear documentation and benchmarks. Government bodies would benefit from the use of accurate data and research to inform guidelines and legislation.

### Strengths and limitations

This is the first systematic review using rigorous methods to evaluate how, when and what physiotherapy services are used by older adults in nursing homes. The results provide insight into current physiotherapy practice in nursing homes internationally, and highlight gaps in knowledge that need to be addressed to ensure effective and equitable physiotherapy services are available for nursing home clients. The PRISMA checklist was used to ensure transparency and accuracy in the reported methods and findings. The use of the Anderson Healthcare Utilisation Model further strengthened this systematic review by highlighting key factors that should be considered in the development and improvement of physiotherapy services in nursing homes [20, 33].

The conclusions of this review are limited by the lack of data provided on the parameters of physiotherapy service, including type, frequency, funding, and monitoring. None of the included studies provided information on physiotherapy assistants, therefore it was not possible to determine to what extent they play a role in nursing homes. The majority of the studies did not provide information on the inclusion criteria and/or the validity and reliability of the measures used. Seven studies used either self-reported surveys or interviews, which could have introduced bias due to over- or under-reporting by participants [21, 25, 27–31]. Three studies used the MDS or RAI assessments, which provide cross-sectional data over a short timeframe (seven days), and potentially lead to inaccurate reporting of regular physiotherapy service use in nursing homes [22, 23, 26]. One study used manual examination of clinical notes [24]. This process could have resulted in missed physiotherapy services due to human error.

In this systematic review there was no time limit applied to the search criteria due to the likelihood there would be a small number of studies on this topic (11 identified in this systematic review). This resulted in the inclusion of papers that spanned over 20 years, which limited the comparisons that can be drawn between papers as the demographics and disease profiles of nursing home residents have changed considerably during this time.

## Conclusions

Emerging evidence suggests physiotherapy services are accessed by some nursing home clients, but specific details on these services are lacking. Factors associated with physiotherapy service use suggest that older adults who may require more support with their care needs due to cognitive disorders, older age or greater length of stay are actually less likely to receive physiotherapy services. It is unclear if effective physiotherapy services are currently used in nursing homes or are available to those at greatest risk of deterioration. Further investigation into the use of physiotherapy services in nursing homes is needed to develop evidence-based physiotherapy benchmarks and standards specific to the needs of older adults that live in nursing homes.

## Supporting information

S1 File Systematic review protocol. (PDF)

S1 Table PRISMA 2009 checklist [14]. (DOC)

S1 Fig Search strategies. (DOCX)

S2 Fig JBI critical appraisal checklist for analytical cross sectional studies [19]. (DOCX)

S3 Fig Data extraction form [19]. (DOCX)
