# Supplementary material for: The use of physiotherapy in nursing homes internationally: A systematic review
Source: PLoS One. 2019 Jul 11;14(7):e0219488. doi: 10.1371/journal.pone.0219488 (PMC6623957; doi:10.1371/journal.pone.0219488)
Supplement: S1 File — (PDF) [file pone.0219488.s001.pdf]

When and how are qualified physiotherapists utilised by older adults living in residential aged care: A systematic review protocol

PROSPERO registration number: CRD42018082460

Reviewers/Authors:

Lindsey Brett

Tim Noblet

Andrew Georgiou

Mikaela Jorgensen

Review question:

When and how are qualified physiotherapists utilised by older adults living in residential aged care?

Specific objectives:

- Provide a descriptive (and analytical) analysis of when and how qualified physiotherapists are utilised by older adults in residential aged care facilities, including aspects such as frequency, duration, funding, type of employment, type of services used and level of care
- Provide a descriptive (and analytical) analysis of how qualified physiotherapist services are monitored, including documentation, outcome indicators used and effectiveness
- Compare qualified physiotherapist provisions in residential aged care facilities internationally
- Where possible, consider the characteristics of older adults living in residential aged care facilities that receive physiotherapy services that involve a qualified physiotherapist (this will depend on the details provided in studies identified for the systematic review)

Background

As the population continues to age there is an increasing number of older adults that require care and assistance with activities of daily living (ADLs). For some this means relocation into residential aged care facilities (RACFs) where they can receive formal care services. Residential aged care facilities are known by various terms worldwide, such as nursing homes, aged care homes and long-term care facilities.[1] For the purpose of this review the term *'residential aged care facilities (RACFs)'* will be used as this is the term commonly used in the Australian healthcare sector. The Australian Institute of Health and Welfare define a RACF as *'a special-purpose facility which provides accommodation and other types of support, including assistance with day-to-day living, intensive forms of care, and assistance towards independent living, to frail and aged residents'*.[2] In Australia, the Aged Care Assessment Team determine approval for Government-subsidised residential aged care through assessment of the care needs of older adults and adults living with a disability.[3] Funding is then determined by a group of assessments conducted by RACF staff that consider

relative care needs related to ADLs, behaviours, and complex health care; collectively known as the Aged Care Funding Instrument (ACFI).[4] As of June 2011 36% of permanent residents were assessed as having high care needs for ADLs, 34% for behaviours, and 25% in the complex health care domain.[3] There are a greater proportion of females living in Australian RACFs (70%), and 57% of permanent residents are aged 85 years and older.[3]

To ensure individual care needs are met, RACFs are serviced by nurses, general practitioners and allied health professionals. One commonly utilised allied health profession is physiotherapy. Physiotherapists currently play an important role in administering ACFI; their assessments contribute to information gathered for the ADLs domain, as well as pain management which is within the complex health care domain.[5] Dependent on the RACF, physiotherapists can also be asked to assist with mobility and movement related dysfunctions, rehabilitation, fall and injury risk minimisation, individualised exercise prescription, incontinence management, and staff manual handling education and training.[6]

Physiotherapists can be utilised to treat musculoskeletal, cardiorespiratory and neurological conditions as well as assist with management of chronic pain and other chronic disorders for people of all ages. Existing research demonstrates the benefits of moderate to high intensity physiotherapy for older people that live in RACFs; improvements have been observed in strength, endurance, balance, physical function, ability to complete ADLs, falls prevention and reduction in pain.[7-11] To treat an individual successfully a physiotherapist will use a range of interventions, which can include but are not limited to physical exercise, education, soft tissue techniques including massage, joint mobilisation and manipulation, electrotherapy (e.g. ultrasound and TENs), prescription of assistive devices, taping and dry needling.[12]

There is legislation worldwide that outlines the required standard of care in RACFs, such as the Aged Care Act (1997)[13] and Quality of Care Principles (2014)[14] in Australia, NICE Guidelines[15] and Care Standards Act (2000)[16] in England, and the Nursing Home Reform Act (1987)[17] in America. Such legislations recommend the use of physiotherapy to help restore and maintain the physical function of older people living in RACFs. Physiotherapy for older people is also promoted worldwide by the various physiotherapy bodies, including the Australian Physiotherapy Association, The Chartered Society of Physiotherapists and the Academy of Geriatric Physical Therapy. However, current legislations do not outline minimum requirements for physiotherapy or other allied health services in RACFs. There is also a variation in how physiotherapy is funded in different countries, and even in different RACFs within the same country, which could influence the provision of physiotherapy. It is not clear how much physiotherapy is received by RACF clients, or the type of interventions they receive. To ensure the care needs of older adults living in RACFs are being adequately addressed, it is essential we have an understanding of when and how physiotherapy is being utilised. Digital health systems could help to address this issue as more RACFs transition to digital health systems.

Using keywords from the research question a preliminary search for previous systematic reviews on this topic was conducted using several databases: Cochrane database for systematic reviews, PubMed, MEDLINE, CINAHL, PROSPERO and JBI database of systematic reviews and implementation

report. No systematic reviews on the when and how physiotherapy is utilised older adults living in RACFs were found.

This proposed review will identify and analyse current research and grey literature to help gain a greater understanding of the provisions of physiotherapy by qualified physiotherapists in RACFs. The findings will also help to identify where the current gaps in knowledge are, and lead to suggestions for future research. This review is of particular importance in Australia, where proposed changes to ACFI in 2018 could potentially include the addition of funding for exercise and use of other allied health professionals.[18] It is important to understand the work that Australian physiotherapists currently do in RACFs, to highlight their importance in this field as well as areas their role can be expanded or focused.

Inclusion criteria

Participants

This review will consider studies that involve older adults; participants aged 65 years and older. Participants of any gender and race, with varied comorbidities and level of care requirements will be included in this review. Participants will predominately be permanent residents at the RACFs, respite residents will not be included.

Intervention

All types of physiotherapy services that involve a qualified physiotherapist will be considered, such as physical exercise, massage and prescription of assistive devices.

Context

This review will focus on studies conducted in RACFs. This will include similar facilities that match the definition outlined above (*Background* section) that may be referred to as a different name in different locations across the world. Other commonly used phrases to describe a RACF include nursing home, aged care home, residential home, care home, rest home and long-term care facility. Different facilities globally can provide different levels of care for older people, all types will be considered.

Type of studies

Both quantitative and qualitative studies will be considered in this review, including grey literature, to ensure all available evidence is considered. The focus of the review is a descriptive analysis of how and when qualified physiotherapists are utilised in RACFs, therefore the type of studies that will be considered include observational, surveys, narrative reviews, reports and case studies. Opinion pieces will not be included. If a study considers other allied health professionals, it will only be included if the results related to physiotherapy can be considered independently.

Search strategy

Initially all titles and abstracts of the identified studies will be checked for eligibility by two reviewers independently. If a study does not have an abstract, the full paper will be reviewed. The two reviewers will then confer to resolve any discrepancies, if an agreement cannot be made a third reviewer will be consulted.

Both published and unpublished studies and reports will be included in this review. An initial search will be conducted on MEDLINE to highlight potential studies using the following keywords: physiotherap*, physical therap*, nursing homes and elderly. The titles and abstracts of studies located in this search will be reviewed for keywords and common text to help build the search strategy for this review.

A detailed search using the identified keywords with appropriate truncations and wild cards will be conducted across several databases, including: MEDLINE via Ovid, PubMed, EMBASE, CINAHL, the Cochrane Library, AMED, Pedro and OTseeker. Database searches will be limited to studies in English and publications from 1997. This timeframe was set as major legislations and frameworks related to residential aged care were established from 1997, including: the Australian Aged Care Act (1997), Australian Quality of Care Principles (2014), the UK Care Standards Act (2000), the Canadian Long-term Care Home Act (2007), and the New Zealand Public Health and Disability Act (2000).

The references of all studies that meet the inclusion criteria then be hand-searched for other potential studies. The content of key journals in physiotherapy and gerontology (e.g. the Journal of Physiotherapy, Age and Ageing and Australian and New Zealand Journal of Public Health) will also be searched for any recent studies that have not yet been indexed in the selected databases, due to the time delay between journal publication and indexing.[19]

Grey literature will also be search as part of this review. Sources of grey literature will include: GoogleScholar, Trove and ProQuest Dissertations and Theses Global. Government and relevant associations' (e.g. Australian Physiotherapy Association and National Aged Care Alliance) reports, censuses, surveys and websites will also be searched for appropriate data.

Assessment of methodological quality

Papers that meet the inclusion criteria for this review will be assessed by two independent reviewers to determine the methodological quality. If an agreement cannot be made between the two reviewers, a third reviewer will be consulted to resolve the issue. Methodological quality will be assessed using the appropriate Joanna Briggs Institute (JBI) Critical Appraisal Tool based on the study design/type of document; for example, the JBI Critical Appraisal Checklist for Studies Reporting Prevalence Data (Appendix 1), and the JBI Critical Appraisal Checklist for Qualitative Research (Appendix 2).

Publication bias will be address by the inclusion of grey literature. Dependent on the characteristics of the studies included, if a metanalysis is possible an appropriate statistical test for publication bias will be conducted.

Data extraction

Data extraction from the selected studies will be completed by two reviewers independently using a standardised form. Prior to data extraction both reviewers will become familiar with the standardised form to ensure consistency when completing data extraction. The two reviewers will review the full text of the selected titles to determine eligibility for this review. Once completed, both reviewers will confer to discuss findings and any missing data. The corresponding author for papers that are found to have missing data will be contacted to clarify the information required. If needed a third reviewer will be consulted to discuss any discrepancies when determining which studies will be included in this review.

As a range of study designs will be included in this review a single data extraction form has been developed and pre-tested based on the key items from the JBI data extraction forms for different types of studies (Appendix 4). Data that will be extracted from all included papers are: study design/type of text, country, setting, participant characteristics (e.g. age, gender, comorbidities), type of intervention(s), year/timeframe, main results/conclusion. There will also be specific items that will be completed dependent on the study design, for example reviewer's conclusion for text and opinion papers, and methodology for qualitative papers.

Data synthesis

A narrative of the findings from the included studies will be provided, along with tables and graphs where appropriate to help present the findings. This descriptive approach will focus on: characteristics of the included studies, the type, duration and frequency of physiotherapy interventions that involve a qualified physiotherapist utilised in RACFs, how qualified physiotherapist services are monitored, and the characteristics of older adults that use physiotherapy services that involve a qualified physiotherapist in RACFs.

Aggregated data will be used to analyse any quantitative data collected from the included studies. Any papers that contain quantitative data will be assessed for heterogeneity using the chi-square test. If possible, the data will be pooled and a meta-analysis completed.

References

1. Roberts K. International aged care: a quick guide. In: Section SP, editor. Canberra: Commonwealth of Australia; 2017.
2. Australian Institute of Health and Welfare. Residential aged care facility: Identifying and definitional attributes Canberra: Commonwealth of Australia; 2010 [Available from: http://meteor.aihw.gov.au/content/index.phtml/itemId/384424.
3. Australian Institute of Health and Welfare. Residential aged care in Australia 2010–11: A statistical overview. Canberra: AIHW; 2012.
4. Australian Government. Aged Care Funding Instrument (ACFI) Reports Canberra: Commonwealth of Australia; 2017 [Available from: https://agedcare.health.gov.au/tools-and-resources/aged-care-funding-instrument-acfi-reports.
5. Australian Government. Aged Care Funding Instrument (ACFI) user guide. In: Health Do, editor. Canberra: Commonwealth of Australia; 2016.
6. Position Statement: Supporting older Australians [press release]. Sydney: Australian Physiotherapy Association, June 2012 2012.
7. Chiodo LK, Gerety MB, Mulrow CD, Rhodes MC, Tuley MR. The Impact of Physical Therapy on Nursing Home Patient Outcomes. Physical Therapy. 1992;72(3):168-73.
8. Frandin K, Gronstedt H, Helbostad JL, Bergland A, Andresen M, Puggaard L, et al. Long-Term Effects of Individually Tailored Physical Training and Activity on Physical Function, Well-Being and Cognition in Scandinavian Nursing Home Residents: A Randomized Controlled Trial. Gerontology. 2016;62(6):571-80.
9. Harada N, Chiu V, Fowler E, Lee M, Reuben DB. Physical Therapy to Improve Functioning of Older People in Residential Care Facilities. Physical Therapy. 1995;75(9):830-8.
10. Tse MMY, Ho SSK. Pain management for older persons living in nursing homes: a pilot study. Pain management nursing : official journal of the American Society of Pain Management Nurses. 2013;14(2):e10-21.
11. Jensen J, Nyberg L, Gustafson Y, Lundin-Olsson L. Fall and Injury Prevention in Residential Care—Effects in Residents with Higher and Lower Levels of Cognition. Journal of the American Geriatrics Society. 2003;51(5):627-35.
12. Miller J. Common physiotherapy treatment techniques Sandgate2017 [updated 19/05/2017. Available from: http://physioworks.com.au/FAQRetrieve.aspx?ID=30872.
13. Australian Government. Aged Care Act 1997. In: Health Do, editor. 112 ed. Canberra: Commonwealth of Australia; 1997.
14. Australian Government. Quality of Care Principles 2014. In: Services DoS, editor. Camberra: Commonwealth of Australia; 2014.
15. National Institute for Health and Care Excellence. Older people in care homes Manchester: NICE; 2015 [updated February 2015. Available from: https://www.nice.org.uk/advice/lgb25/chapter/Introduction.
16. UK Government. Care Standards Act 2000. London: National Archives; 2000.
17. Klauber M. The 1987 Nursing Home Reform Act Washington: AARP; 2001 [updated February 2001. Available from: https://www.aarp.org/home-garden/livable-communities/info-2001/the_1987_nursing_home_reform_act.html.
18. Rosewarne R, Opie J, Cumpston R, Boyd V, Kikkawa A. Review of the Aged Care Funding Instrument report, Part 1: Summary report. Balwyn: Applied Aged Care Solutions Pty Ltd; 2017.
19. Armstrong R, Waters E, Jackson N, Oliver S, Popay J, Shepherd J, et al. Guidelines for Systematic reviews of health promotion and public health interventions. Australian: Melbourne University; 2007 October 2007.
