# Supplementary material for: The use of physiotherapy in nursing homes internationally: A systematic review
Source: PLoS One. 2019 Jul 11;14(7):e0219488. doi: 10.1371/journal.pone.0219488 (PMC6623957; doi:10.1371/journal.pone.0219488)
Supplement: S1 Fig — (DOCX) [file pone.0219488.s003.docx]

**S1 Fig. Search Strategies**

Database and grey literature searches were optimised by using terms identified through an initial search on MEDLINE.

**DATABASES**

**Databases: MEDLINE via Ovid and EMBASE**

(physiotherap* or physical therap*) and (nursing homes or residential aged care* or long?term care* or homes for the aged or residential facilit* or aged care* or rest homes or care homes or long?term residential care* or personal care homes or institutional care* or skilled nursing facilit* or group homes or assisted living facilit* or intermediate care facilit*) and (aged or geriatrics or elder* or old* or ag?ing)

*Limits applied:* English language and full text and yr=”1997-Current”

**Database: PubMed**

((((physiotherap* OR physical therap*)) AND residential facilities[MeSH Terms]) AND aged[MeSH Terms]) AND English[Language]

*Limits applied:* full text and yearr=”1997-Current”

**Database: CINAHL**

(physiotherap* or physical therap*) and (nursing homes or residential aged care* or long#term care* or homes for the aged or residential facilit* or aged care* or rest homes or care homes or long#term residential care* or personal care homes or institutional care* or skilled nursing facilit* or group homes or assisted living facilit* or intermediate care facilit*) and (aged or geriatrics or elder* or old* or ag#ing)

*Limits applied:* English language and full text and year=”1997-2018”

**Database: AMED**

(physiotherap* or physical therap*) and (nursing homes or residential aged care* or long?term care* or homes for the aged or

residential facilit* or aged care* or rest homes or care homes or long?term residential care* or personal care homes or institutional care* or skilled nursing facilit* or group homes or assisted living facilit* or intermediate care facilit*) and (aged or geriatrics or elder* or old* or ag?ing)

*Limits applied:* English language and full text and year=”1997-Current”

**Database: Cochrane Library**

(physiotherap* or physical therap*) and (nursing homes or residential aged care* or long?term care* or homes for the aged or

residential facilit* or aged care* or rest homes or care homes or long?term residential care* or personal care homes or institutional care* or skilled nursing facilit* or group homes or assisted living facilit* or intermediate care facilit*) and (aged or geriatrics or elder* or old* or ag?ing)

*Limits applied:* 1997-2018

**Database: Pedro**

Search 1:

*Abstract & title:* "nursing homes" physiotherap*

*Subdiscipline:* Gerontology

*Since:* 1997

Search 2:

*Abstract & title:* "nursing homes" physical therap*

*Subdiscipline:* Gerontology

*Since:* 1997

**Database: OTseeker**

Search 1:

"nursing homes" physical therap*

Search 2:

"nursing homes" physiotherap*

**GREY LITERATURE**

**Database: Google Scholar**

("physiotherapy intervention" OR "physical therapy intervention") "nursing homes" prevalence, elderly

*Limits applied:* English, 1997-2018

**Database: ProQuest Dissertations & theses global**

(physiotherap* OR "physical therap*") AND ("nursing homes" OR "residential aged care*" OR "long?term care*" OR "homes for the aged" OR "residential facilit*" OR "aged care*" OR "rest homes" OR "care homes" OR "long?term residential care*" OR "personal care homes" OR "institutional care*" OR "skilled nursing facilit*" OR "group homes" OR "assisted living facilit*" OR "intermediate care facilit*") AND (aged OR geriatrics OR elder* OR old* OR ag?ing)

*Limits applied:* after 1996, full text
