# Supplementary material for: The use of physiotherapy in nursing homes internationally: A systematic review
Source: PLoS One. 2019 Jul 11;14(7):e0219488. doi: 10.1371/journal.pone.0219488 (PMC6623957; doi:10.1371/journal.pone.0219488)
Supplement: S2 Fig — (DOCX) [file pone.0219488.s004.docx]

**S2 Fig. JBI Critical Appraisal Checklist for Analytical Cross Sectional Studies [19].**

**JBI Critical Appraisal Checklist for Analytical Cross Sectional Studies**

Reviewer Date

Author Year Record Number

|  | Yes | No | Unclear | Not applicable |
| --- | --- | --- | --- | --- |
| 1. Were the criteria for inclusion in the sample clearly defined? | □ | □ | □ | □ |
| 1. Were the study subjects and the setting described in detail? | □ | □ | □ | □ |
| 1. Was the exposure measured in a valid and reliable way? | □ | □ | □ | □ |
| 1. Were objective, standard criteria used for measurement of the condition? | □ | □ | □ | □ |
| 1. Were confounding factors identified? | □ | □ | □ | □ |
| 1. Were strategies to deal with confounding factors stated? | □ | □ | □ | □ |
| 1. Were the outcomes measured in a valid and reliable way? | □ | □ | □ | □ |
| 1. Was appropriate statistical analysis used? | □ | □ | □ | □ |

Overall appraisal: Include □ Exclude □ Seek further info □

Comments (Including reason for exclusion)
