# Supplementary material for: The use of physiotherapy in nursing homes internationally: A systematic review
Source: PLoS One. 2019 Jul 11;14(7):e0219488. doi: 10.1371/journal.pone.0219488 (PMC6623957; doi:10.1371/journal.pone.0219488)
Supplement: S3 Fig — (DOCX) [file pone.0219488.s005.docx]

**S3 Fig. Data extraction form [19].**

[**Data extraction form**](https://reviewersmanual.joannabriggs.org/display/MANUAL/Chapter+5+Appendix+-+Data+extraction+form+for+prevalence+studies)

**Citation Details**

| Authors |  |
| --- | --- |
| Title |  |
| Journal |  |
| Year |  |
| Volume (Issue): Pages |  |
| Record number |  |

**Study details**

| Study design |  |
| --- | --- |
| Country |  |
| Data collection period |  |
| Aim |  |
| Setting/Context |  |
| Inclusion & exclusion criteria |  |
| Measurement method |  |
| Validity and reliability |  |
| Data analysis |  |
| Physiotherapy characteristics |  |
| Client characteristics |  |
| RACF characteristics |  |
| Main findings (n) |  |
| Bias/limitations |  |
| Other comments |  |

**Extraction of findings complete? Yes No**
